# Creolin in Gonorrhœa

**Published:** 1889-05

**Authors:** 


CREOLIN IN GONORRHŒA.—Gonorrhœa, which has resisted other treatment, has frequently yielded in Dr. Margaretti's practice to irrigations, twice daily, with a solution of Creolin of the strength of 5 to 8 per cent. administered through a hollow sound.—*Lancet.*

